# What ails medical research in Pakistan? Role of institutions

**DOI:** 10.12669/pjms.316.9337

**Published:** 2015

**Authors:** Mohammad Perwaiz Iqbal

The tremendous advancement in medical research in USA owes much to the Flexner’s report in 1910 which suggested making the scientific research an essential component of medical curriculum.^1^ National Institute of Health (NIH) of the United States during 1946-50, put the biomedical research as its top priority and faculty was encouraged to implant the “virus of curiosity and inquiry” very early in the curriculum.^2^

Backed by a strong political will, medical research blossomed in that country. Many countries around the world adopted this course and have developed a strong research base. Unfortunately in Pakistan, research in general, and medical research in particular, has been the low priority area for the Government of Pakistan. Pakistan Medical Research Council (PMRC) was established more than 50 years ago with the main tasks to organize, coordinate and promote medical research within the country and advise the Federal and Provincial Governments on all matters related to medical research and healthcare.^3^ In spite of financial constraints, it did make some contribution towards developing guidelines for certain national committees and programs, conducted National Health Survey of Pakistan, provided small research grants (Rs. 200,000) and started Pakistan Journal of Medical Research in 1958 which has yet to get indexed in PubMed. Its scientific personnel include only 14 PhD scholars.^3^ On the other hand, Indian Council for Medical Research (ICMR) has 26 national institutes and 6 regional research centers to address health problems of the country and it publishes a prestigious Indian J Med Res with a reasonable impact factor.^4^ Dearth of physician scientists is a global problem and of greater magnitude in Pakistan because of lack of monetary incentives for medical graduates to get attracted to the PhD path. While there are a few physician scientists in various institutes of PMRC, ICMR has addressed this issue by offering Emeritus Scientists positions to retired medical scientists thereby enabling them to continue research on various medical projects.^4^

Being affiliated with the Ministry of Health, PMRC could have played a more proactive role in addressing Millennium Development Goals (MDGs): number 4 (reduce child mortality of under-five-year-old by two thirds by 2015), number 5 (reduce maternal mortality by three quarters and provide access to universal reproductive health) and number 6 (combat HIV/AIDs, malaria and tuberculosis etc.), in Pakistan. However, for a wide variety of reasons including meagre funding and subnormal capacity, it could not attain the role of a premier institution for the conduct of high quality medical research to address various national health problems.

Another body which was supposed to play a major role in the promotion of medical research in the country was the Pakistan Medical and Dental Council (PMDC) which had the authority for effective regulation of medical education within the country.^5^ Because of politicization of this august body, it has been marred with controversies and its executive council has been dissolved by the Government of Pakistan on the directions of the Supreme Court in August 2015. There have been allegations of allowing the registration of scores of private medical colleges despite their poor infra-structure. It is widely believed that some of its previous council’s decisions have seriously affected the standard of medical education and research within the country. It could not address some of the challenges of the new Millennium. Foremost among these challenges was the introduction of a medical curriculum which is in line with the modern concepts of teaching and learning – a curriculum which imparts the traits of critical thinking, prepares students to be the problem solvers and sensitizes them in early years to research so that they could further develop the spirit of scientific curiosity. This cannot be achieved without recruiting internationally acclaimed teaching faculty with distinctive training in medical research. For the past several decades most medical colleges in the country had excellent clinical faculty, however, recruitment of highly qualified basic sciences faculty has remained a challenge because of an acute dearth of MBBS-PhD scholars within the country.

Thirty two years ago the Aga Khan University, the first private University in Pakistan, took a bold initiative of recruiting basic science faculty to teach medical students. Majority of the faculty members did not have a basic degree in medicine; however, they had excellent training in medical research and several years of experience of teaching in medical colleges in North America and Europe. That paid dividends and many of the graduates of this University have been holding key academic leadership positions within the country and abroad. In all academic rankings of universities in Pakistan by the Higher Education Commission (HEC), this University has enjoyed top position in healthcare research for the last several years. Keeping in view the example set by this University and some other factors, PMDC, about two decades ago, relaxed its insistence on recruiting only MBBS faculty for basic science teaching, and for several years basic science teaching in medical colleges in Pakistan was carried out by PhDs with excellent training in basic medical sciences such as Anatomy, Biochemistry, Physiology and Pharmacology. However, in August, 2012 PMDC took a decision not to accord recognition to basic science faculty with PhD if they did not have MBBS degree. It was a retrogressive step which was not in line with the ground realities because the country had only a few MBBS-PhD scholars in those disciplines. To make the matter worse, PMDC allowed MBBS-MPhil scholars to rise to the level of full professor. It has accorded recognition to nearly 90 Pakistani journals and only a few of these journals have been indexed in PubMed. This facilitated promotion of the faculty without PhD in medical colleges to higher ranks with few publications in peer-reviewed and indexed journals, while promotion to the rank of Assistant Professor in most international universities would not be possible without a PhD and excellence in the scholarship of research. Many universities in Pakistan started churning out MBBS-MPhil graduates without paying much attention to the quality of their research training in medicine. Sensing the possible ill-effects of the PMDC’s decision on medical research within the country, HEC floated a proposal to PMDC to recruit PhDs without MBBS to cover the dearth of PhDs with MBBS. As per report of Mr. Asif Chaudhry in *Dawn*, August 6, 2012, PMDC strongly turned down that proposal on the ground that “such professionals were irrelevant to the medical field”.^6^ Unfortunately, that inflexible attitude still persists with some of the council members who remain oblivious of the fact that there is an acute dearth of MBBS-PhD scholars even in the developed countries and that shortage has been addressed most judiciously by recruiting experts in medical science disciplines whether or not they had a basic degree in medicine. While medical research has been flourishing in those developed countries and some of our neighboring countries, it has not made a significant impact on the body of scientific knowledge at the international level. Apart from a couple of medical universities, the research output of most medical colleges in Pakistan presents a dismal picture.

A survey conducted to assess the attitudes and practices of postgraduate medical trainees towards research clearly indicated poor research training and insufficient awareness about research as the major reasons for poor state of medical research in Pakistan while most trainees had positive attitudes towards health sciences research.^7^ This shows that most medical graduates do recognize the importance of research, however, lack of research infrastructure and paucity of medical scientists as mentors are their major concerns which require immediate attention.

It is expected from the PMDC to take stock of the situation of basic science teaching and research in various medical colleges in Pakistan and adopts a pragmatic approach. Many talented basic scientists who had gone abroad to excellent universities on HEC scholarships for PhD are returning to Pakistan. This is a precious human resource. They should be accommodated in medical colleges on the basis of their quality of research training in medicine and experience of teaching in medical schools/universities of international repute rather than getting hung-up on a basic degree in medicine. They should be provided an enabling environment and an opportunity to make their contribution towards the uplift of medical research within the country by inculcating traits of critical thinking among medical students and junior faculty, developing their ability to formulate research questions and devising feasible work plans, thereby equipping them with sufficient research skills and knowledge to embark on addressing various national health problems.

In a recent publication on this subject, a host of recommendations have been suggested to medical institutions to promote research culture in Pakistan, which include appointments and promotions of faculty strictly on the basis of academic and research accomplishments, formation of research-support units in all medical institutions, recognition of distinguished researchers at the Annual Research Day/Convocation and allocation of funds to faculty on the basis of research output and contribution towards capacity building, especially on developing the research skills of human resources.^8^ It is time to seriously consider the above mentioned recommendations and implement them in true letter and spirit in order to prepare this country for future challenges to healthcare.
